# Comparison of digital panoramic radiography versus cone beam computerized tomography for measuring alveolar bone

**DOI:** 10.1186/s13005-017-0135-3

**Published:** 2017-02-22

**Authors:** Zunan Tang, Xianchu Liu, Kejia Chen

**Affiliations:** 1 Department of Oral Radiology, Xiangya Stomatological Hospital, Central South University, No. 72 Xiangya Road, Changsha, 410078 China; 2 School of Stomatology, Central South University, Changsha, China

**Keywords:** Cone beam computerized tomography, Digital panoramic radiography, Dental implanting, Magnification rate

## Abstract

**Background:**

Cone beam computerized tomography (CBCT) has been widely used in dental implanting. However, the local hospitals usually don’t have access to CBCT due to the cost and medical investment, especially in West of China. The doctors in local hospitals have to make reasonable dental planting using orthopantomography (OPG) to reduce risks. Therefore, it is clinically meaningful to determine the magnification rate of OPG to obtain correct diagnosis. This study investigated the magnification rate of OPG in measuring different maxillofacial loci compared with CBCT.

**Methods:**

Eighty-six patients demanding dental implanting were scanned by CBCT and OPG. The vertical distance between the alveolar ridge crest of the maxillary first molar and the sinus bottom of the upper jaw, the distance between the alveolar ridge crest of the mandibular first molars and the top of nerviduct in the mandibular alveolar, and the distance between the alveolar ridge crest of the maxillary central incisors and the bottoms of the nasal cavities were measured. The horizontal distance in those loci were also measured. The distances derived from CBCT were used as reference. The distances between the two methods were compared using paired t-test. The magnification rates at these positions were calculated. The relationship between the data acquired from the two methods was analyzed Pearson correlation.

**Results:**

The correlation coefficients (R) between the paired samples obtained from OPG and CBCT were highly related (*P* < 0.05) with R values varying from 0.840 and 0.959 in vertical distances and R values varying from 0.703 and 0.904 in horizontal distances. Compared with data obtained from CBCT, the mean vertical magnification rates were 11.38% and 12.95% vertically and 8.55% and 9.43% horizontally for the first molars in the right and left maxilla respectively; 7.26% and 6.35% vertically and 5.33% and 4.96% horizontally for the first molars in the right and left mandible respectively; and 5.55% and 4.84% vertically and 6.53% and 7.47% horizontally for the central incisors in the upper right and left jaws respectively.

**Conclusion:**

The magnification rates of OPG at these teeth are different. The distances measured by OPG were highly correlated with that measured by CBCT.

## Background

Panoramic radiography and periapical radiography are important methods in dental implant planning [1, 2]. However, these two-dimensional radiographs can be affected by tissue superimposition due to malocclusion deformity or other complex situations. Orthopantomography (OPG) is an important imaging method to assess vertical bone volume and detect dental caries and periodontal diseases [3]. OPG has many advantages including panoramic, easy and cheap to conduct, and informative regarding jaw morphology, bone density, etc. Therefore, OPG is one of the most common imaging methods for routine examination in clinical practice [4–6].

In recent years, maxillofacial cone beam computed tomography (CBCT) has been widely used in dental implanting [7, 8], assessment of orthodontic treatment, complex alveolar surgery, oral local system reconstruction and treatment of tooth and dental pulp diseases. CBCT is advantageous in high spatial resolution, short scan time and rapid image acquisition [9–13].

With the improvement of living standards, the demand for dental implantation is increasing quickly. In China, it is a trend to use three dimensional imaging of high precision to replace two-dimensional imaging for stomatological diagnosis. However, due to the cost and medical investment, the local hospitals usually don’t have access to CBCT, especially in West of China. The doctors in local hospitals have to make reasonable dental planting using OPG to reduce risks. Therefore, it is clinically meaningful to determine the magnification rate of OPG to obtain correct diagnosis. Then, clinicians could be able to estimate the real bone measurement based on the OPG measurement and the magnification rate to obtain the ideal implants placement. In the present study, the data of OPG images and CBCT images from 86 patients demanding dental implanting were compared and analyzed. The main endpoints were the magnification rates of OPG in measuring the vertical and horizontal distance at different maxillofacial loci.

## Methods

### Patients

The study was approved (reference number: 20150902) by the Medical Ethics Committee of Xiangya Stomatological Hospital of Central South University and the signed informed consent was obtained from each patient. Eighty-six patients (48 male, 38 female; 15-67 years old, mean age of 41.3), who demanded for dental implantation and had OPG and CBCT in Xiangya Stomatological Hospital of Central South University between July 2013 and September 2014, were enrolled in the study. The inclusion criteria were the patients with clear OPG and CBCT images showing the bottoms of nasal cavity, maxillary sinus and inferior alveolar nerve tube, and the patients with normal jaw and good periodontal condition. The exclusion criteria were medical history of jaw lesions or jaw surgery, severe malocclusion deformity or artifacts resulted from metal denture.

### Scanning methods

For OPG scanning, the patients were in standing position with cervical spine staying vertically. The lower jaw was placed in the middle of the chin support. The incisal edge of the front teeth bit on the groove of the plate. The head was perpendicular to the ground. The angle bisector between the orbitomeatal line and acanthiomeatal line was parallel to the ground. OPG was performed using a digital panoramic scanner (Planmeca, Promax, Finland), with the voltage of 66 kV, current of 9 mA and the minimum exposure time of 16 s. The data were saved in JPG format.

For CBCT scanning, the patients were in sitting position with cervical spine staying vertically. The sagittal laser line of the CBCT scanner (Planmeca, Promax 3D Max, Finland) was superimposed with median sagittal line of the patients. The lower jaw was placed in the middle of the chin support. Their upper and lower dentitions were at centric occlusion position. The occlusion was parallel to the ground. The patients held the handle for automatic CBCT scanning. During the scanning, the mouth kept motionless. CBCT was operated at the voltage of 96 kV, current of 10 mA with scanning field of 10 × 9 cm using matrix of 512 × 512. The scan time was 15 s, the data was stored in Dicom format and the images were reconstructed using slices of 0.15 mm and measured. All scans were performed by the radiologists with more than 3 years working experience. For the same patients, OPG and CBCT were taken on the same day.

### Measurement methods

For the vertical measures, the distances (a and b) between the alveolar ridges of bilateral maxillary first molars (16, 26) and the bottoms of the maxillary sinus, the distance (c and d) between the alveolar ridges of bilateral mandibular first molars (36, 46) and inferior alveolar nerve canal, and the distance (e and f) between the alveolar ridge crest of bilateral maxillary central incisors (11, 21) and the bottoms of the nasal cavities were measured. As for the horizontal measures (the implants width), the followings were measured: the distances (g and h) between the alveolar ridges of the adjacent teeth of maxillary first molars (16, 26), the distance (i and j) between the alveolar ridges of the adjacent teeth of mandibular first molars (36, 46) and the distance (k and l) between the alveolar ridges of the adjacent teeth of maxillary central incisors (11, 21). The tomographic volumes were manually measured and analyzed with Planmeca Romexis 3.0.1 R workstation. All measurements were taken twice by two experienced radiologists independently and the mean values were used for analysis. The interclass correlation co-efficiency (ICC) was used as evaluating indicators to determine the reliability of the same observer and between observers.

**Fig. 1:**
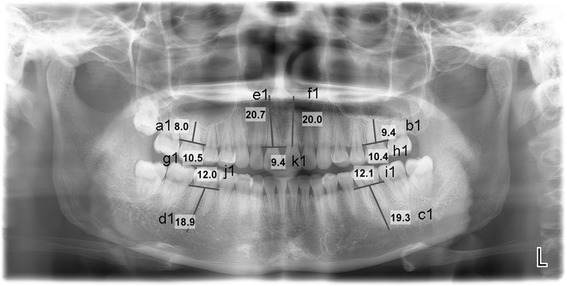
OPG image showing the measurement sites

For OPG, the midpoints between mesial and distal alveolar ridges were used as the measuring points to measure the distances a1, b1, c1, d1, e1 and f1 (Fig. 1). The distance between the mesial (15) and distal (17) alveolar ridges of the adjacent teeth of maxillary first molars (16) was measured as g1. The distance between the mesial (25) and distal (27) alveolar ridges of the adjacent teeth of maxillary first molars (26) was measured as h1. The distance between the mesial (35) and distal (37) alveolar ridges of the adjacent teeth of mandibular first molars (36) was measured as i1. The distance between the mesial (45) and distal (47) alveolar ridges of the adjacent teeth of mandibular first molars (46) was measured as j1. The distance between the mesial (12, 21) alveolar ridges of the adjacent teeth of maxillary central incisors (11) was measured as k1. The distance between the mesial (11, 22) alveolar ridges of the adjacent teeth of maxillary central incisors (21) was measured as l1.

**Fig. 2:**
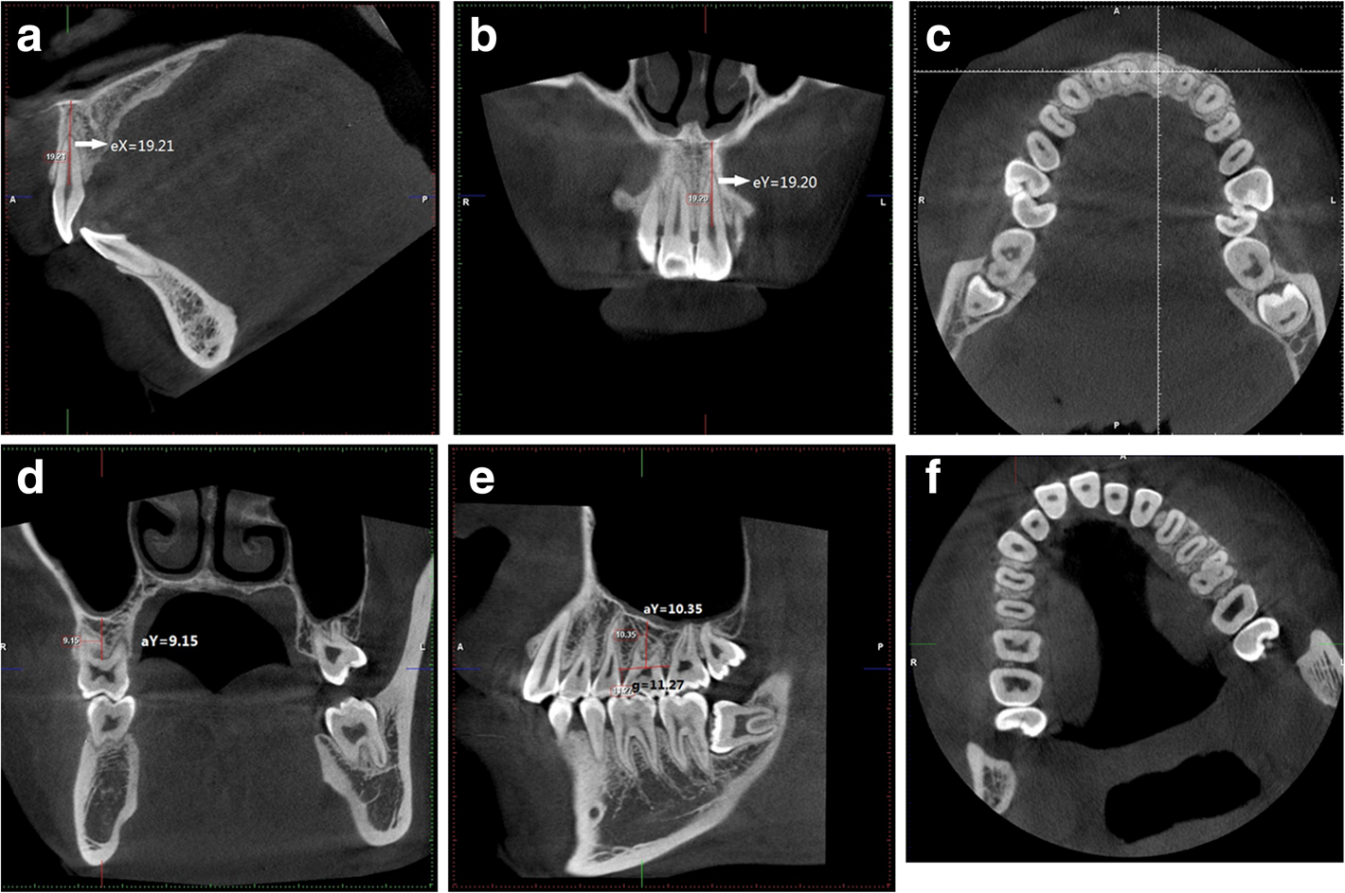
CBCT images showing the vertical measurement on X **a**, Y **b** and Z **c** planes and horizontal measurement **d**-**f**

For CBCT, the reconstructed images were rotated on the plane X (sagittal plane) and plane Y (coronal plane) to superimpose the long axis of the tooth with the observational vertical scale bar. The images were rotated on the plane Z (cross-sectional plane) to make the tangent of the dental arch superimposed with the sagittal plane, and the mesial-distal tooth line superimposed with the coronal plane. The line distances a-X, a-Y, b-X and b-Y were measured from the alveolar ridge crests of the teeth 16 and 26 to the bottoms of the maxillary sinus on the planes X and Y respectively. The distances a2 and b2 were determined: a2 = (aX + aY)/2 and b2 = (bX + bY)/2. Similarly, c2 (c2 = (cX + cY)/2), d2 (d2 = (dX + dY)/2), e2 (e2 = (eX + eY)/2), and f2 were measured (Fig. 2). On the plane X, the midpoint of the mesial-distal alveolar ridge line was used as the measuring points of the alveolar ridge. On the plane Y, the midpoint between the buccolingual and alveolar ridge was used as the measuring points of the alveolar ridge. The horizontal distance of g2, h2, i2, j2, k2 and l2 were measured on the CBCT images.

The magnification formula was defined as magnification rate = (a1- a2)/a2 × 100%.

### Statistical analysis

All data were analyzed using SPSS 19.0 (SPSS Inc, Chicago, USA). The paired t-test was used to compare the vertical distances and horizontal distance between the two methods. *P* < 0.05 was considered statistically significant. The normality of data was tested using the k-w test. Pearson correlation analysis was used to analyze the relationship between the data acquired using the two methods. The correlation coefficient (R) between the paired samples was calculated and was considered highly related if R was between 0.5 and 1.

## Results

The ICC value of the repeated measurements by the same observer was 0.97 and 0.95 respectively and the ICC value of the measurements by different observers was 0.93. The ICC values were above 0.8.

**Fig. 3:**
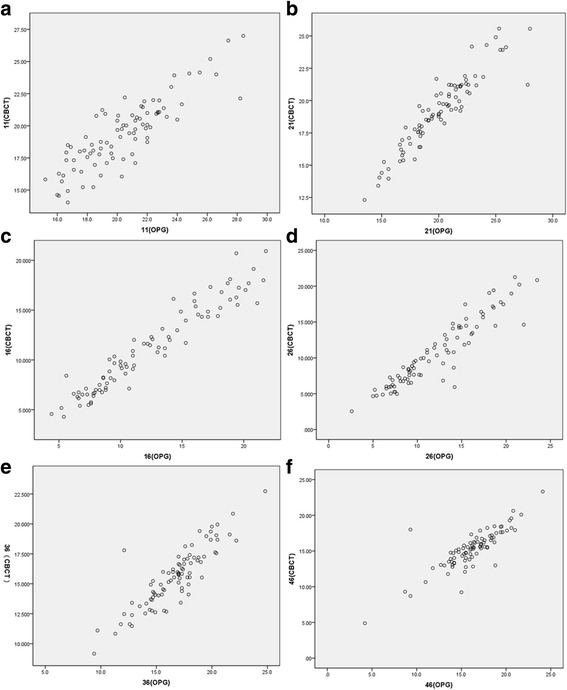
The scatter plot showing the correlations between the two sets of the vertical measurements. The correlations in in the teeth of 11 **a**, 21 **b**, 16 **c**, 26 **d**, 36 **e** and 46 **f**

**Fig. 4:**
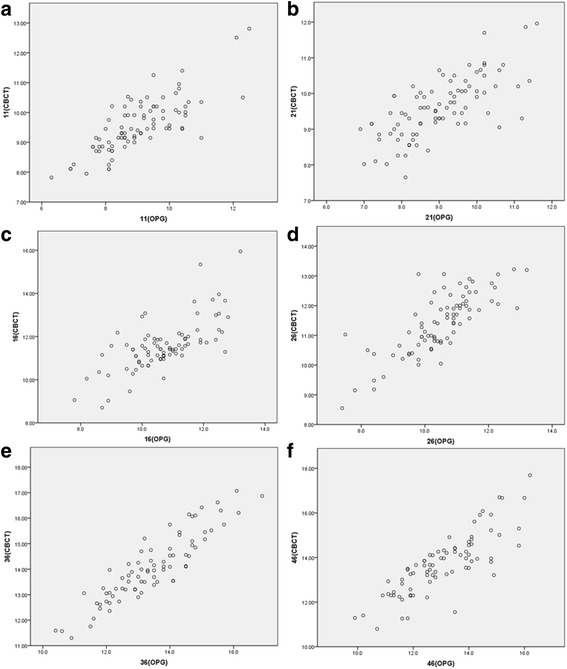
The scatter plot showing the correlations between the two sets of the horizontal measurements. The correlations in the teeth of 11 **a**, 21 **b**, 16 **c**, 26 **d**, 36 **e** and 46 **f**

The correlation coefficients (R) between the paired samples obtained from OPG and CBCT were highly related (*P* < 0.05) with R values varying from 0.840 and 0.959 in vertical distances at different teeth and R values varying from 0.703 and 0.904 in horizontal distances (Table 1). The correlations between the two sets of data are shown in Figs. 3 and 4. Compared with the data obtained from CBCT, the mean vertical magnification rates were 11.38% and 12.95% vertically and 8.55% and 9.43% horizontally for the first molars in the right and left maxilla respectively; 7.26% and 6.35% vertically and 5.33% and 4.96% horizontally for the first molars in the right and left mandible respectively; and 5.55% and 4.84% vertically and 6.53% and 7.47% horizontally for the central incisors in the upper right and left jaws respectively.

**Table 1:** Correlation coefficients between the paired samples obtained from OPG and CBCT at different teeth

Teeth	11	21	16	26	36	46
CC in vertical distance	0.858*	0.930*	0.959*	0.933*	0.880*	0.840*
CC in horizontal distance	0.777*	0.707*	0.703*	0.783*	0.904*	0.828*

* indicates a significant correlation (*P* < 0.05)

*CC* correlation coefficient, *OPG* orthopantomography, *CBCT* cone beam computerized tomography

## Discussion

The present study showed different magnification rates of OPG compared with CBCT in measuring different maxillofacial loci. There were highly related correlation coefficients (R) between the paired samples obtained from OPG and CBCT.

In CBCT reconstructed 3D images, the morphology of alveolar ridge and the height of alveolar bone can be accurately displayed, [14, 15] showing buccolingual thickness, mesiodistal width, clear local bone structures and their anatomical relationship with surrounding anatomical structures, especially inferior alveolar nerve tube and the maxillary sinus. These images can assist to determine the volume of the bone, and the position, direction and volume of the implants, which are of great value for pre-implanting planning[16, 17]. However, due to high technical requirements and high cost, CBCT is not available in many local hospitals in China. OPG is widely used in local hospitals [2, 18], but it is hard to precisely determine the height of alveolar bone and the relationship between maxillary sinus and inferior alveolar nerve tube. The image is largely affected by the body position with variable magnification rates and distortion rates for different parts [19, 20]. Previously, panoramic radiography is shown to be a sufficiently accurate method to obtain the interatral bone height in the incisor area, but not in the canine area as compared with CBCT [21].

It is meaningful to determine the magnification rate of OPG for clinical guidance. The present study showed that the vertical OPG magnification rates of the first molars in the right and left upper jaws were 10.83% and 13.02% respectively; the magnification rates of the mandibular first molars were 7.09% and 5.96% respectively. The correlation coefficients (R) between the paired samples obtained from OPG and CBCT in the vertical and horizontal direction were highly related (*P* < 0.05). Therefore, the magnification rates could possibly provide some guidance for implanting when determining the safety distance based on OPG images [22, 23].

Distortion of panoramic radiographs may be resulted from the distance between the X-ray source, and film or imaging plate, the difference between the axis motion track of scan and the shapes of inspected parts, and the velocity of film (or imaging plate) relative to the X-ray beam[20, 24, 25]. The distortion rate may differ due to instruments, shooting positions and measurement methods, and is therefore very different, typically ranging from below 30% to over 50% [26, 27]. Besides, the form and symmetry of the dental arch, teeth arrangement, teeth shape, the tilt angle of teeth and the surrounding tissues also exert an influence on the image. Tong et al. found that the vertical OPG magnification rate is about 25%, which is the same as the magnification rates of the machine [28]. He also showed that the magnification rate is similar among the patients, with few exceptions. These results are consistent with ours.

The mean vertical magnification rates were 5.55% and 4.84% for the central incisors in the upper right and left jaws respectively, suggesting that OPG is relatively accurate for measurement of vertical bone volume. The mean vertical magnification rates were 6.53% and 7.47% for the central incisors in the upper right and left jaws respectively; the difference is influenced by the shape of alveolar bone, rotation angle and tilt angle of the teeth. The factors affecting the measurement of the front tooth are mainly the inclination angle of the alveolar bone, nasal shape and position. The major factors limiting the planting of front tooth are the faciolingual thickness of bone plate and OPG as two-dimensional imaging is not able to provide information regarding the thickness. Therefore, we didn’t perform any comparison concerning the thickness between the two scanning methods, which is a limitation of our study. During the clinical practice, CBCT has to be used when the alveolar bone density and thickness in the implanting area couldn’t be determined by OPG. There are other limitations. For example, the medial lateral nerve location and the avoidance of structures had better to be taken into consideration. The comparison at more loci should be further performed.

## Conclusion

During our clinical practice, we found that there was huge difference in alveolar bone volumes obtained from OPG and CBCT in the patients with periodontal diseases. Lin et al. reported that the alveolar bone density decreased in the patients with periodontitis, which was in consistent with the changes of their alveolar bone.[29] Therefore, these patients were excluded from the study. To lower the risks, patients with moderate or severe periodontal diseases are not suggested to implant based on OPG alone. In conclusion, the magnification rates of OPG at these teeth are different. The distances measured by OPG were highly correlated with that measured by CBCT.

## Abbreviations

CBCT: Cone beam computerized tomography
ICC: Interclass correlation co-efficiency
OPG: Orthopantomography
